# Multiple Models of European Marine Fish Stocks: Regional Winners and Losers in a Future Climate

**DOI:** 10.1111/gcb.70149

**Published:** 2025-04-03

**Authors:** Sévrine F. Sailley, Ignacio A. Catalan, Jurgen Batsleer, Sieme Bossier, Dimitrios Damalas, Cecilie Hansen, Martin Huret, Georg Engelhard, Katell Hammon, Susan Kay, Francesc Maynou, J. Rasmus Nielsen, Andrés Ospina‐Álvarez, John Pinnegar, Jan Jaap Poos, Vasiliki Sgardeli, Myron A. Peck

**Affiliations:** ^1^ Plymouth Marine Laboratory (PML) Plymouth UK; ^2^ Mediterranean Institute for Advanced Studies (IMEDEA, CSIC‐UIB) Esporles Spain; ^3^ Wageningen Marine Research Wageningen University and Research IJmuiden the Netherlands; ^4^ Technical University of Denmark, National Institute for Aquatic Resources Kongens Lyngby Denmark; ^5^ Nippon Foundation Ocean Nexus, School of Resource and Environmental Management (REM) Simon Fraser University (SFU) Burnaby British Columbia Canada; ^6^ Hellenic Centre for Marine Research (HCMR) Anavyssos Greece; ^7^ Institute of Marine Research (Havforskningsinstituttet) (IMR) Bergen Norway; ^8^ DECOD (Ecosystem Dynamics and Sustainability) IFREMER, INRAE, Institut Agro Brest France; ^9^ Centre for Environment, Fisheries and Aquaculture Science (Cefas) Lowestoft UK; ^10^ Wageningen Economic Research Wageningen University and Research Wageningen the Netherlands; ^11^ Department of Coastal Systems Royal Netherlands Institute for Sea Research (NIOZ) Den Burg (Texel) the Netherlands

**Keywords:** climate change, fish stock, fishery, model ensemble, regional change, species distribution

## Abstract

Climate change continues to alter the productivity of commercially and culturally important fisheries with major consequences for food security and coastal economies. We provide the first, multi‐model projections of changes in the distribution and productivity of 18 key fish stocks across seven European regional seas spanning the Mediterranean to the Arctic, using 11 state‐of‐the‐art bio‐ecological models. Our projections indicate species‐ and region‐specific changes in abundance and distributions of these stocks by the mid‐ to late 21st century. The varied responses are caused by differences in species' physiology, regional food web dynamics, and physical habitat characteristics. Important drivers include not only warming of Europe's seas (from 1°C to 3°C in RCP 4.5, and 2°C to 4°C in RCP 8.5 by 2100) and changes in primary productivity but also oxygen‐limited fish growth, changes in pH, and benthic dissolved organic carbon. Warming and altered levels of secondary production are projected to lead to declines in some stocks (Norwegian and Barents Sea herring) and increases in others (Bay of Biscay anchovy). While some temperate and cold‐water stocks are projected to decline markedly in some regions (e.g., North Sea, Western Mediterranean), the immigration of species from the south and/or increase in productivity of warm‐water species may offer new opportunities for fisheries. Species‐level changes will likely have ecosystem‐level consequences that have yet to be fully assessed, and responses in some sub‐areas may be more pronounced due to local processes not captured in projections. Projections are consistent despite differences in model structures, and the results of our multi‐model analysis align with other modelling exercises while delving into details often overlooked at the species or spatial level. This represents a novel approach to projecting the impacts of climate change on fisheries, which should be considered in future efforts to support climate‐ready management strategies for marine fish stocks.

## Graphical abstract

Using state‐of‐the art models we projected changes for 17 key commercial fish species in the Northeast Atlantic under two climate scenario. Carrying the analysis at the species level and regional scale rather than the usual total biomass approach, we identify “winners and losers” for fish and regional fisheries, highlighting the need for novel approach for climate ready management strategies for marine fish stock.

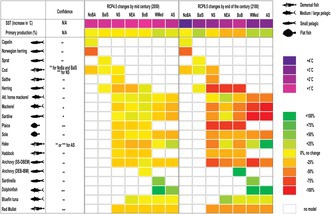

## 1 Introduction

Marine fisheries provide important fundamental and demand‐derived ecosystem services (Holmlund and Hammer 1999). This is especially true in the European Union, the world's largest single market for fish and fish products (FAO 2022). A primary goal of the EU is to effectively manage its fish stocks and sustainably grow its aquaculture sector to promote self‐sufficiency in the domestic supply of fish and seafood (European Commission et al. 2017). However, climate change poses risks and offers potential opportunities to these goals, both for EU marine fisheries and worldwide. Historical analyses highlight clear shifts in the distribution of commercially important fish stocks in regional seas on both sides of the North Atlantic (Baudron et al. 2020; Nye et al. 2009; Peck and Pinnegar 2018; Perry et al. 2005; Pinsky et al. 2013; Rijnsdorp et al. 2009) associated with changes in temperature and other physical as well as biogeochemical factors. Various modeling exercises have projected how fish stocks will respond in a future climate (Peck et al. 2018; Plagányi 2007) and numerous estimates are available at the global level (Cheung et al. 2018; Lotze et al. 2019; Maltby et al. 2020). These global analyses suggest that warming will shift the distribution of marine species leading to net gains (temperate) and losses (equatorial) in community diversity (Garciá Molinos et al. 2016; Lotze et al. 2019) while the global decrease in primary production, change in primary producer phenology or species composition, and/or shift in the timing of events will lead to reduced standing fish stock biomass (Barange et al. 2018; Pinsky et al. 2013; Worm and Lotze 2021).

Hotspots of climate‐driven warming have been detected in European regional seas (Hobday and Pecl 2014), where important differences exist in the dominant bottom‐up factors shaping habitat suitability for fish, such as dissolved oxygen, salinity, pH, and lower trophic level productivity. Despite the relative affluence of European countries, climate‐driven changes in fish stocks pose high economic and social risks to some coastal fishing communities (Payne et al. 2020). A good example is the North Sea Cod ( *Gadus morhua* ) with the impact of warming on reproduction (O'Brien et al. 2000) and distribution (Engelhard et al. 2014) as well as studies on the effect of ocean acidification on the larvae and recruitment of juveniles (Frommel et al. 2012; Stiasny et al. 2016). Therefore, tailored regional assessments are required to understand historical changes and to make robust projections of climate change impacts on fish stocks, their ecosystems, and the services they provide. Considering that the effects of climate change are not spatially or temporally uniform in the world's oceans, examining regional‐scale changes is important for planning climate adaptation strategies (Peck and Pinnegar 2018). While the usual management unit uses national Exclusive Economic Zones (EEZs), the changes that affect fish stocks are not limited to these areas. Thus, this study focuses on the effect of climate change at the larger scale of European regional seas. We are also examining various valuable species rather than fishing stocks or total fish biomass. The responses of species can then be compared at the basin scale to provide a mixed‐fisheries perspective on the impact of climate change. This additionally allows us to identify changes requiring adaptation measures by regional fisheries, such as shifts in fish community composition or displacement of fishing grounds, which are challenging to detect using traditional metrics such as changes in overall potential catch and/or fish biomass. This approach aims to provide a more complete picture of the future of fisheries than standard stock assessments and is the first to leverage multiple models to focus on the location and health of stocks. It also expands on the common EEZ approaches that emphasize potential catch and/or total fish biomass (Lotze et al. 2019; Tittensor et al. 2021).

## 2 Materials and Methods

### 2.1 Lower Trophic Level Model: Projected Physical and Biogeochemical Changes in European Seas

The fish stock projections discussed here are forced with outputs from regional‐scale physical and biogeochemical models of the European seas (Kay 2018; Table S1). The best (at the time of the work) available model projections of physical and biogeochemical changes were used. Due to regional differences among areas, the Norwegian and Barents Seas used ROMS, the Baltic Sea used HBM‐ERGOM, and all other regions used POLCOMS ERSEM (Table S1 and citations therein for details on the models). The projections from the latter model can be found on the Copernicus Climate Data Store (Kay 2020). The regional models were driven by global models from the fifth Coupled Model Intercomparison Project (CMIP5) and used two Representative Concentration Pathways (RCPs) of greenhouse gas concentrations. In the moderate scenario, RCP 4.5, CO_2_ levels rise until the mid‐21st century and then stabilize; in the more extreme RCP 8.5, levels continue to rise throughout the century (van Vuuren et al. 2011). All models output variables such as salinity, sea surface temperature (SST), nutrients, chlorophyll (total or per phytoplankton groups), primary production (PP), and zooplankton biomass. Each model used the necessary outputs to project the impacts on fish (see Table S1 for the reference to each models and which outputs they use).

**FIGURE 1:**
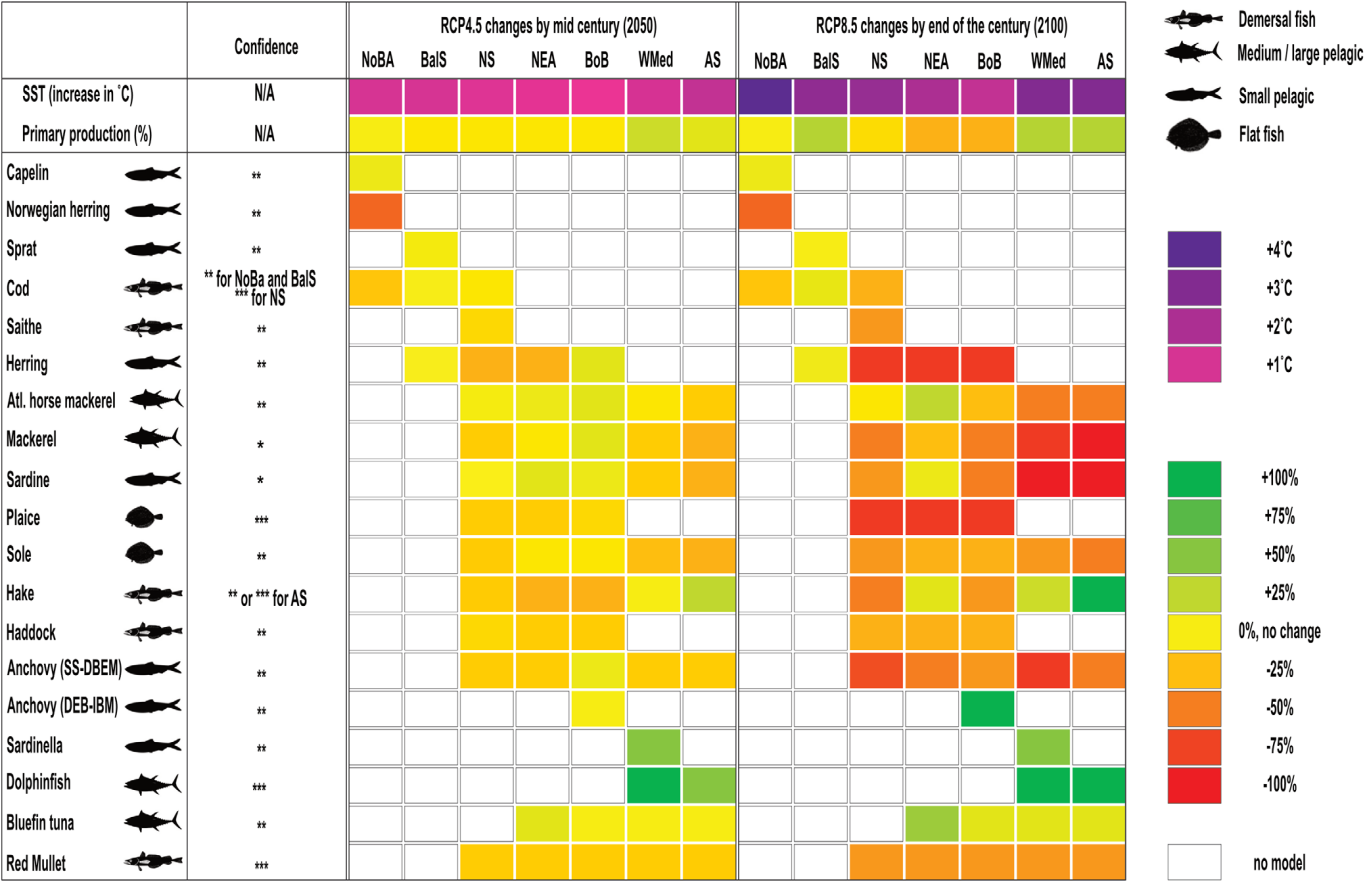
Qualitative overview of the projected change in sea surface temperature (SST in °C; scale from light to dark purple), primary production (PP, % change to average PP in 2000–2010), and fish species (% change compared to average biomass in 2000–2010). For numerical values, see Table S3. The color scale for PP and fish biomass/growth changes ranges from −100% (red) to +100% (green) with yellow as the mid‐point value. Confidence in model outputs (see Section 2) yielded scores lacking some bounding categories, so here are expressed as low (*), medium (**) and high confidence (***) reflecting level of agreement between model runs, number of models used in the area, and historical trends. AS, Aegean Sea; BalS, Baltic Sea; BoB, Bay of Biscay; NEA, Northeast Atlantic; NoBA, Norwegian and Barents Seas; NS, North Sea; WMed, Western Mediterranean. Note that the confidence in the biogeochemical models used for SST and primary production was not assessed (N/A).

The main variable having an impact on fish and being used by all models was SST and PP. SST warming of 0.5°C–2.0°C and 1.0°C–3.0°C is projected under RCP 4.5 and RCP 8.5, respectively, for most European seas, with larger increases for the Barents Sea (Figure 1). Primary production (PP) is projected to decrease by up to 10% in most areas but to increase by about 20% in the Mediterranean Sea (Figure 1). Depending on the fish model, a climatology of the time slice was used, or the models were run continuously with monthly or annual outputs. While analyzing results, we observed only modest differences projected between RCP 4.5 and 8.5 by mid‐century, as well as between mid‐ and end‐century under RCP 4.5. This pattern was consistent across SST, PP, and the responses of fish species (for models running continuously from 2000 to 2100 with monthly or annual resolution). Therefore, we present results from RCP 4.5 for mid‐century (2050–2060) and RCP 8.5 for the end of the century (2090–2100) to better emphasize the changes while avoiding redundancy.

### 2.2 Higher Trophic Level Model Selection

**FIGURE 2:**
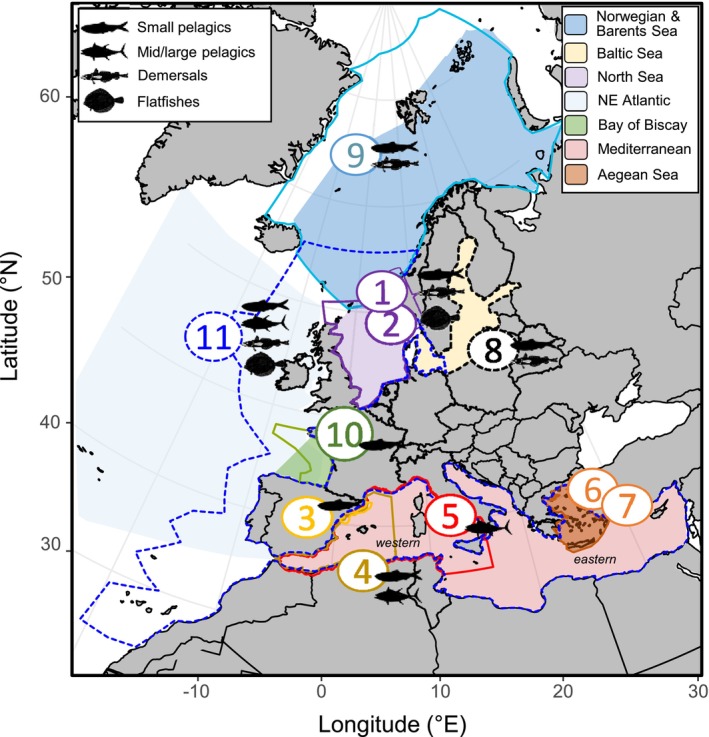
Basins or sub‐basins (color‐shaded areas) and model domains (contours and numbers) referred to in Table S1. Models are statistical, mechanistic, or mixed. Statistical models are: 1, INLA; 2, SDM‐habitat; 3 and 6, SDM‐GAM; 4 and 5, Spawning models; 7, SDM‐MaxEnt. Mechanistic models are: 8 and 9, Atlantis; 10, IBM. Number 11, SS‐DEBM, is a mixed statistical‐mechanistic model (see text). Note that model 11 includes NE Atlantic and Mediterranean. Fish silhouettes correspond to broad fish groups (see Section 2 for detailed species information). Underlying coupled physical‐biogeochemical models are presented in Table S1. Map lines delineate study areas and do not necessarily depict accepted national boundaries.

We selected 17 species that are important to EU industrial fisheries, along with one targeted by a small‐scale fishery, the dolphinfish (see Figure 1 or Table S2 for the full list). Mechanistic models were applied to knowledge‐rich species, while statistical approaches were employed for species with more limited data (see Table S2 for the correspondence between fish species and models). Models ranged from statistical (Damalas et al. 2021; Maynou et al. 2020; Moltó et al. 2021; Peck et al. 2020; Phillips et al. 2006; Rambo et al. 2022; Reglero et al. 2019; Rue et al. 2009; Townhill et al. 2023; Zuur et al. 2017) or coupled bio‐physical life cycle models (Bossier et al. 2018) for single species to complex, end‐to‐end models of food web interactions (Bossier et al. 2018, 2021; Fernandes et al. 2020; Hansen et al. 2016, 2019; Figure 2; Tables S1 and S2). With a few exceptions, projections were made using two models for most species and regional seas including the Norwegian and Barents Seas (NoBa), NE Atlantic (NEA), North Sea (NS), Baltic Sea (BalS), Bay of Biscay (BoB), western Mediterranean Sea (WMed), and Aegean Sea (AS). A mechanistic model considers aspects of the ecology (e.g., habitat preference and migration) and physiology (e.g., growth and reproduction) to determine biomass and distribution of fish species in response to changes in the environment (e.g., temperature, competition with other species, food availability). On the other hand, the statistical models use historical data to find the relationship between environmental factors (e.g., temperature and primary production) and species (e.g., abundance of a key life cycle stage) or processes (e.g., larval survival, growth of juveniles/adults, distribution); they then apply these relationships to climate change projections and assess change compared to present‐day conditions. The mechanistic models are multi‐species or single species, while the statistical models are single species models that can be applied to more than one species. Model results provided either change in population biomass or individual growth, which we compared for model agreement for the change in biomass per species per region (Figure 1; Table S3).

The models differed in structural complexity due to regional differences in available data, knowledge on the target species/system, as well as differences in the questions, the models were originally designed to address. The main caveat is that some models have a specific focus and cannot be extended beyond that. For example, the multi‐species models (SS‐DBEM model 11, NoBa model 9 and Baltic Atlantis model 8; Table S1) can represent a large number of fish species and include some but not all of the potential complexities in the life cycle dynamics of those species (e.g., potential stage‐specific recruitment bottlenecks driven by climate change). On the other hand, the single species, 0D‐DEB‐IBM (model 10; Bay of Biscay anchovies; Table S1), or the INLA model (model 1; North Sea plaice; Table S1) provide rich detail in the seasonal dynamics of various life stages. However, no single tool can adequately represent the potential climate change—driven processes impacting early life stages and recruitment dynamics while, at the same time, including critical interactions among fish species (e.g., competition for resources, predation, and prey switching) that may also shift in unexpected ways due to climate change. Despite their different focuses, all models looked at the impact of climate change on one or multiple fish, with biomass being the most common output.

Another caveat was the implementation of fishing impact across the models. While the impact of fisheries is not negligible and, in some cases, can make stocks less resilient to climate change, only some of the models (models 8 to 11, Table S1) could run scenarios of both climate change and fishing. Furthermore, in some cases, it was not possible to vary the degree of fishing pressure due to large uncertainties in historical or current fishing mortalities experienced by fish or due to limitations in model structure. Consequently, we do not compare the impact of fishing on fisheries. For further details on the models, see the referenced literature for each in Table S1.

### 2.3 Estimation of Confidence in Models

An agreement was observed in the changes projected by different models that overlapped in their domain and target species. The confidence in the model outputs (Figure 1, confidence column) was assessed using the IPCC methodology, which evaluates agreement between the model and evidence supporting it (Figure S1).

The agreement was scored based on projections for the same species and region, considering both the direction and strength of the trend. For cases where one model was applied, scoring was dependent on whether multiple scenarios were explored and whether the model was state‐of‐the‐art or newly implemented. This approach evaluated agreement within the “ensemble” of projected models. Evidence was scored based on the extent of agreement with existing validation data (historical and present‐day field data) and past model validation exercises, where available.

Both agreement and evidence were scored on a scale from 1 (poor) to 3 (very good). When there was uncertainty, the lower score was chosen. The scores were multiplied to generate a final confidence score ranging from 1 to 9, categorized into five levels: very low (1+), low (2+), medium (3+), high (6+), and very high (9) (Peck et al. 2020). This approach mirrors the IPCC methodology (See Figure S1 for scoring and confidence scale, Table S5 for individual scores).

It is important to note that confidence in the physical and biogeochemical models was not assessed because all models (1) projected mid‐range climate impacts (i.e., average warming trend compared to other model projections within the CMIP products that are warmer or colder), avoiding extreme responses; (2) were previously validated; and (3) are related to CMIP products included in IPCC‐type assessments.

## 3 Results

As mentioned, using climate projections, we forced a suite of 11 models producing projections for specific species or sets of species, both demersal and pelagic, in either a single region or across several regions.

### 3.1 Impact on Fish Abundance and Location

The overall productivity and biomass of the 18 fish species examined were projected to decline (Figure 1) within European waters under RCP 4.5 (≥ 15% decrease by 2050) and RCP 8.5 (≥ 40% decrease by 2100). We found consistent results for the direction of change in population biomass, growth, or distribution of the same species in the same region across different models. This is specifically reflected in the confidence scoring, particularly in the agreement component (Figure 1; Table S5). The intensity of the response to changes in SST and PP, however, was region‐specific (e.g., increases were projected for several species in the WMed and AS) and species‐specific (e.g., changes ranged from −30% to 10% for species in the BoB under RCP 4.5). The combination of warmer conditions and reductions in food availability will, in most cases, decrease the diversity and productivity of key commercial fish species. On the other hand, the increase in primary production and, hence, food availability projected for the WMed and AS suggests that fish species less sensitive to warming (i.e., with a broader temperature tolerance or higher preferred or tolerable temperature; Pörtner and Peck 2010) would fare well or even potentially thrive in the future (e.g., dolphinfish and round sardinella), if properly managed and not overharvested.

**FIGURE 3:**
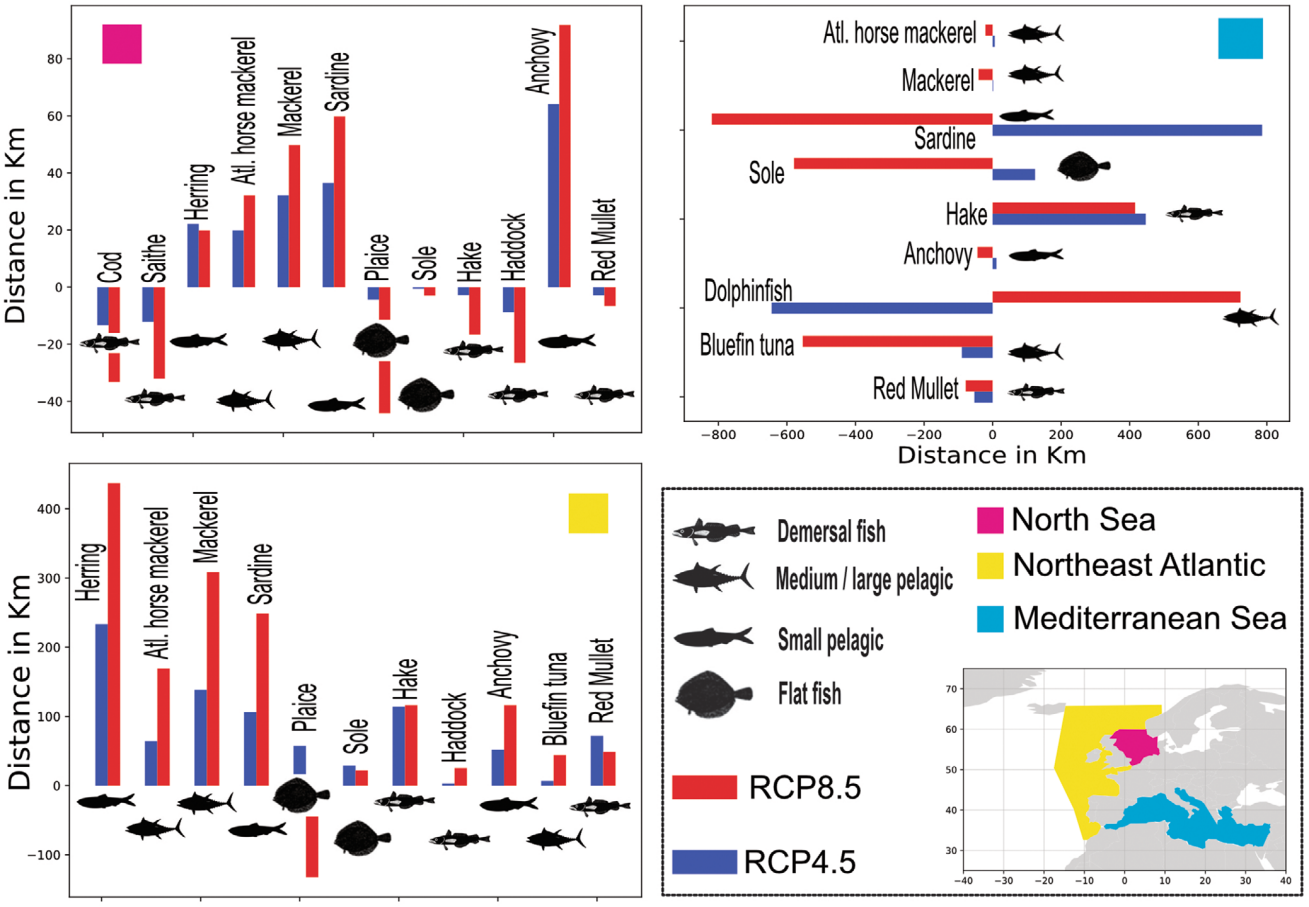
Change in the location of the fish population center by the end of the century compared to 2000–2010, per basin expressed in km, in a no fishing scenario (See also Table S4). Note that we are only showing the main direction of change, which is North–South in the NE Atlantic and North Sea, while it is East–West in the Mediterranean Sea. Results from the SS‐DBEM were used to calculate the change in population centroid. Map lines delineate study areas and do not depict accepted national boundaries.

While the change in a species' biomass in a specific region is important for the potential catch, changes in fish distribution could be more critical to fisheries in terms of time at sea and catch‐to‐effort ratio. Using the model with the largest domain and the most species (SS‐DBEM, model 11, Figure 2), we calculated changes in population centroid (i.e., the center of the population distribution) in the NS, NEA, and the Mediterranean Sea (Figure 3; Table S4). In the NEA, all species (except plaice) shifted to higher latitudes, and the shift was more pronounced under RCP 8.5 than RCP 4.5. The change in centroid between RCPs ranged from 3 to 25 km for haddock (under RCP 4.5 and 8.5, respectively; Table S4) and from 230 to 430 km for herring (under RCP 4.5 and RCP 8.5, respectively; Table S4). Conversely, in the NS, we observed a diverging trend in the change in the centroid between pelagic (e.g., herring, sardine, and horse mackerel) and demersal (e.g., place, sole, hake, and haddock) species shifting to higher and lower latitudes, respectively. The difference in these directions was due to the need for demersal species to inhabit continental shelf waters and the reduction in the spatial extent of optimal habitats in the NS. Finally, in the Mediterranean Sea, the change in centroid location was along an east–west gradient, with most of the fish species increasing in the western part of their distribution. When combined with the change in biomass, the direction of the shift suggested species expansion/improvement (dolphinfish, tuna; Moltó et al. 2021; Peck et al. 2020) or eventual extirpation (European anchovy; Townhill et al. 2023; see also Gkanasos et al. 2021; Maynou et al. 2020) with changes in centroid location ranging from 2 to 786 km in RCP 4.5 (tuna and dolphinfish, respectively; Table S4) and 20 to 800 km under RCP 8.5 (red mullet and dolphinfish, respectively; Table S4). This illustrates why looking at both changes in biomass and shifts in the location of stocks is important for understanding not only the impact of climate change but also its impact on fisheries and local communities.

While the overall trend is toward a biomass decrease and a shift toward more suitable habitats (i.e., following isotherms; Burrows et al. 2011), as seen in other works (Cheung et al. 2016; Dahms and Killen 2023; Engelhard et al. 2014), changes projected for species in the NoBa and BS were relatively small (Norwegian herring aside, with a 60% decline, the changes ranged from +5% to −20%). For the NoBA region, only modest warming was projected, and trophic interactions were found to be more relevant (Hansen et al. 2019). On the other hand, while the BalS was projected to experience significant warming under RCP 8.5, the climate‐driven changes in fish stocks were negligible (compared to other European Seas). This is because the BalS is an enclosed basin where long‐term eutrophication (Murray et al. 2019) has played a larger role in PP and, by extension, fish production.

Important stocks of pelagic (herring), demersal gadoids (cod, saithe, hake, haddock), and flatfish (plaice) exist in the NS, a region that offers contrasts in projected change depending on the species (Figure 1). Under RCP 4.5 and RCP 8.5, all these stock biomass was projected to decrease from 10% to 20% and 10% and 80%, respectively, by the end of the century. While the loss of some species (hake and haddock) was less severe under RCP 4.5 than RCP 8.5, sharp declines in other species (herring, cod, plaice and saithe) occurred under both RCPs. In both RCPs, SST was projected to warm by on average 2°C and PP to decrease by 5%. The reduction of suitable habitat was particularly severe for demersal species for which limits in depth distribution constrain their ability to shift to deeper waters at higher latitudes to maintain present‐day temperatures (Petitgas et al. 2013). This explains why the centroid of some species shifted to lower latitudes (Figure 3) with projected habitat compression (Engelhard et al. 2011; Jorda et al. 2019). In line with expectations (Petitgas et al. 2012; Schickele et al. 2020), the distribution of several pelagic species was projected to shift to higher latitudes.

In contrast to latitudinal shifts (Figure 3), projections for Mediterranean Sea fish stocks suggested some longitudinal shifts and maintenance of present‐day levels of abundance in a future climate. The suite of species examined in the WMed offers interesting contrasts in projected responses. Traditional targets of small pelagic fisheries (sardine and anchovy) that display historically low levels of spawning stock biomass in recent decades were projected to decline under RCP4.5 and (for commercial purposes) collapse under RCP 8.5 (Figure 1; Maynou et al. 2020; Peck et al. 2020). Conversely, our projections show enhanced habitat suitability for the tropical to sub‐tropical sardinella, as found elsewhere (Sabatés et al. 2006), which may partly compensate for the loss of traditional target species. On the other hand, little or no change was projected in either RCP 4.5 or RCP 8.5 for bluefin tuna driven by improved larval survival in this key spawning area (Reglero et al. 2019). Finally, dolphinfish supports important artisanal fisheries (Moltó et al. 2020) and was projected to be a “climate winner”. Under both RCPs, this thermophilic species was projected to spawn earlier, grow faster, and temporally increase its presence, leading to a longer fishing season (Moltó et al. 2021; Rambo et al. 2022), although abundance changes were not estimated. In the AS, the habitat suitability for two commercially important demersal species (hake and red mullet) was projected to increase in two separate models under both RCPs, potentially leading to increased catches (Damalas et al. 2021).

Aside from the BoB, no subdivision was considered for the NEA. Throughout the NEA, the trend was toward a displacement of the different fish species toward higher latitudes with dramatic declines in the abundance of thermally sensitive species such as mackerel and herring. Although the region was projected to maintain present‐day levels of fish abundance under RCP 4.5, poleward shifts on the order of 138 and 233 km for mackerel and herring, respectively, were projected. Under RCP 8.5, shifts were larger (308 and 437 km, respectively). Consequently, some sub‐areas within the NEA were projected to have increases or decreases in commercially important fishes, with some areas gaining new species and others losing traditional fisheries targets. Projections for species in the Bay of Biscay highlight the importance of spatial scale, with some species showing trends that are opposite or of different amplitude to that registered at the broader scale of the NEA.

## 4 Discussion

The outcomes of our projections are consistent with other large‐scale modeling exercises (Peck and Pinnegar 2018; Tittensor et al. 2021) reporting a reduction of fish biomass and poleward migration of fisheries targets. However, previous modeling efforts often compromise on certain details, such as species resolution (e.g., focusing on single species, multispecies with some level of aggregation, or total biomass and/or catch potential) or the spatial resolution (e.g., one basin, one ocean, or global studies often simplify to the EEZ level; Lotze et al. 2019; Tittensor et al. 2021). In this multi‐model exercise, we achieved a balance by analyzing individual species across relevant scales, including basin and regional. We believe that our approach allowed us to identify specific opportunities and risks. This is exemplified by the findings that several small pelagic or demersal species exhibited a future decline in some regions and an increase in other regions (see Figure 1). This is even more evident when considering that species that show a decline in some areas might be increasing in others due to displacement of the population centroid (see Table S4). A caveat to the study is that we used only one “consensus” physical and biogeochemical model to provide the necessary forcing; other models might have provided different amplitudes to the change in drivers or directions of trend which would alter the results of the fish models we used. One such case is the Mediterranean Sea, where projections in primary production are arguable. However, some authors (Moullec et al. 2019) project a generalized increase in fish biomass in the Mediterranean Sea (in this sense, agreeing with the current results) but much larger in the Eastern than in the Western Mediterranean. Another potential issue is in some cases the lack of full food web interaction within the fish model. For example, the SS‐DBEM projects an increase in bluefin tuna and dolphinfish but a decrease in anchovies and sardines, their main prey based on the size‐spectrum theory that indicates primary production is enough to sustain enough fish at all size classes, but not whether the right preys are available. This is a concern in any model that deals with food webs. However, it does not take away from our result as there is a general agreement with other models that used other physical and biogeochemical models or even multiple ones.

It is important to note that our projections focused on species that currently play important commercial, cultural, and/or ecological roles in specific systems. Although the fisheries in some regions would be projected to be overall ‘climate losers’ in our analyses (e.g., those relying on traditional fisheries targets in the NS and southern portion of the NEA), vessels and gears targeting temperate or cold‐water fishes may be able to catch warmer‐water species that increase in the future. This will require successful climate adaptation such as adopting new gear designs, gaining experience fishing “new” species, and developing new markets (Pinnegar et al. 2016). Such increases can be driven by the immigration of new species via poleward migration (Garciá Molinos et al. 2016; IPCC 2014; Lloret et al. 2015; Tsikliras and Stergiou 2014) or increased local productivity of species historically present (Petitgas et al. 2012). The dolphinfish in the Mediterranean Sea is an example of the latter process. Most current projection tools are not designed to explore ecosystem‐level impacts of shifts in species composition and, thus, whether these changes exacerbate climate impacts or increase climate resilience is unclear.

Although our projections focused on SST and primary production, these are not the only drivers of change in fish stocks. For example, climate‐driven changes in secondary production (i.e., zooplankton) were projected to be a key factor in European anchovy in the BoB and Norwegian spring spawning herring. Analyses conducted on fish stocks in the AS indicated that pH and benthic dissolved organic carbon were additional important drivers. Similarly, a temperature increase will raise the metabolic demands of fish, which may be impossible to meet at low oxygen levels (Catalán et al. 2019). Although current mechanisms are vigorously debated (Audzijonyte et al. 2020), deoxygenation could be a more important process than climate‐driven warming (Pörtner et al. 2017) and demands further study. Additional gaps in knowledge stem from the general lack of spatially explicit regionalized food web models capable of exploring trophic amplification of biomass loss (Kwiatkowski et al. 2019). While climate change is predicted to be an important driver of changes in fisheries resources, climate‐ready fisheries management has an important role to play (Free et al. 2020; Woods et al. 2022) as overfishing will exacerbate declines in traditional fish stocks, and plans will be needed for the sustainability of “new” stocks. This would include tools that can harness fishers' perspectives on how patterns of exploitation may respond to future changes in the abundance and distribution of historical and new target species. Additionally, management strategies need to acknowledge that stocks are not restricted to their country EEZ and should consider the potential of stocks in and out of these boundaries. Wherever possible, cross‐boundary management between countries should be encouraged for stocks that occur across multiple EEZ.

The present study takes an important step forward toward the ideal approach of projecting climate impacts on regional fish stocks by using multiple biological models forced with high‐resolution regional climate information, an approach long discussed (Jones et al. 2013; Stock et al. 2011) but not yet implemented in most regional seas. Comparing such projections, understanding why they may differ, and identifying their limitations is paramount to providing the best possible science‐based advice for ongoing global assessments (IPCC 2014), including those performed by the Intergovernmental Panel on Climate Change (IPCC) and the Intergovernmental Science‐Policy Platform on Biodiversity and Ecosystem Services (IPBES). We argue that robust projections from regional models can provide the best underpinning to planning actions to successfully meet key policy objectives such as the United Nations Sustainable Development Goals (SDGs). We believe our work represents the first example of a necessary shift in assessing regional impacts of climate change on multiple stocks. To address critical concerns such as food security, employment, and the cultural significance of fisheries, we recommend that future efforts prioritize the development of multi‐model, regional projections of fish stocks as a global imperative.

## Author Contributions

**Sévrine F. Sailley:** conceptualization, formal analysis, methodology, writing – original draft, writing – review and editing. **Ignacio A. Catalan:** data curation, writing – original draft, writing – review and editing. **Jurgen Batsleer:** data curation, writing – review and editing. **Sieme Bossier:** data curation, writing – review and editing. **Dimitrios Damalas:** data curation, writing – review and editing. **Cecilie Hansen:** data curation, writing – review and editing. **Martin Huret:** data curation, writing – review and editing. **Georg Engelhard:** writing – review and editing. **Katell Hammon:** data curation, writing – review and editing. **Susan Kay:** data curation, writing – review and editing. **Francesc Maynou:** data curation, writing – review and editing. **J. Rasmus Nielsen:** data curation, writing – review and editing. **Andrés Ospina‐Álvarez:** data curation, writing – review and editing. **John Pinnegar:** data curation, writing – review and editing. **Jan Jaap Poos:** data curation. **Vasiliki Sgardeli:** data curation. **Myron A. Peck:** project administration, writing – original draft, writing – review and editing.

## Conflicts of Interest

The authors declare no conflicts of interest.

## Supporting information


Data S1.

## Data Availability

The code for the core models are available from Zenodo at https://doi.org/10.5281/zenodo.7548113 (SS‐DBEM) and https://doi.org/10.5281/zenodo.7300564 (POLCOMS‐ERSEM) with their outputs available from the Copernicus Climate Change Services (C3S) and Climate Data Store (CDS) with the following DOI: https://doi.org/10.24381/cds.39c97304 (SS‐DBEM) and https://doi.org/10.24381/cds.dcc9295c (POLCOMS‐ERSEM). For the other 10 models used to project species distribution, growth, or biomass used in this study link to code and outputs can be found in Table S1.
