# Supplementary material for: Multiple Models of European Marine Fish Stocks: Regional Winners and Losers in a Future Climate
Source: Glob Chang Biol. 2025 Apr 3;31(4):e70149. doi: 10.1111/gcb.70149 (PMC11966357; doi:10.1111/gcb.70149)
Supplement: Supplementary file 1 — Data S1. [file GCB-31-e70149-s001.zip › gcb70149-sup-0002-TableS3.docx]

|  | Confidence | RCP4.5 changes by mid century (2050) | | | | | | | RCP8.5 changes by end of the century (2100) | | | | | | |
| --- | --- | --- | --- | --- | --- | --- | --- | --- | --- | --- | --- | --- | --- | --- | --- |
|  |  | NoBA | BalS | NS | NEA | BoB | WMed | AS | NoBA | BalS | NS | NEA | BoB | WMed | AS |
| SST (increase in ˚C) | - | 1 | 1 | 0.8 | 0.7 | 0.5 | 1 | 1.5 | 4 | 2.7 | 2.5 | 2 | 1.5 | 3 | 3 |
| Primary production (%) | - | 0 | -10 | -10 | -10 | -10 | 20 | 10 | 0 | 30 | -13 | -30 | -30 | 30 | 30 |
| Capelin | ** | 5 | x | x | x | x | x | x | 5 | x | x | x | x | x | x |
| Norwegian herring | ** | -60 | x | x | x | x | x | x | -60 | x | x | x | x | x | x |
| Sprat | ** | x | 2 | x | x | x | x | x | x | -0.6 | x | x | x | x | x |
| Cod | ** (NoBa) ** (BalS) *** (NS) | -23 | -1 | -10 | x | x | x | x | -23 | 7.7 | -30 | x | x | x | x |
| Saithe | ** | x | x | -15 | x | x | x | x | x | x | -40 | x | x | x | x |
| Herring | ** | x | -2 | -30 | -30 | 10 | x | x | x | 3.8 | -80 | -80 | -80 | x | x |
| Atlantic horse mackerel | ** | x | x | 1 | 5 | 10 | -10 | -20 | x | x | -10 | 25 | -25 | -50 | -50 |
| Mackerel | ** | x | x | -20 | -10 | 10 | -20 | -30 | x | x | -50 | -25 | -50 | -80 | -100 |
| Sardine | ** | x | x | -5 | 10 | 5 | -20 | -30 | x | x | -40 | 5 | -50 | -100 | -100 |
| Plaice | *** | x | x | -20 | -20 | -15 | x | x | x | x | -80 | -80 | -80 | x | x |
| Sole | ** | x | x | -20 | -10 | -10 | -25 | -30 | x | x | -40 | -30 | -30 | -40 | -50 |
| Hake | **  *** (AS) | x | x | -20 | -30 | -30 | 0 | 25 | x | x | -50 | 10 | -40 | 20 | 100 |
| Haddock | ** | x | x | -15 | -20 | -20 | x | x | x | x | -30 | -30 | -30 | x | x |
| Anchovy (SS-DBEM) | ** | x | x | -20 | -20 | 5 | -20 | -20 | x | x | -70 | -50 | -40 | -80 | -50 |
| Anchovy (0d-DEB-IBM) |  | x | x | x | x | 0 | x | x | x | x | x | x | 100 | x | x |
| Sardinella | ** | x | x | x | x | x | 50 | x | x | x | x | x | x | 50 | x |
| Dolphinfish | **** | x | x | x | x | x | 100 | 50 | x | x | x | x | x | 100 | 100 |
| Bluefin tuna | ** | x | x | x | 10 | 0 | 0 | 0 | x | x | x | 40 | 10 | 10 | 10 |
| Red Mullet | *** | x | x | -20 | -20 | -20 | -20 | -20 | x | x | -40 | -40 | -40 | -40 | -40 |
