# Supplementary material for: Multiple Models of European Marine Fish Stocks: Regional Winners and Losers in a Future Climate
Source: Glob Chang Biol. 2025 Apr 3;31(4):e70149. doi: 10.1111/gcb.70149 (PMC11966357; doi:10.1111/gcb.70149)
Supplement: Supplementary file 1 — Data S1. [file GCB-31-e70149-s001.zip › gcb70149-sup-0001-Supinfo.docx]

**Supplementary information**

**The supplementary information contains:**

**Figure S1.** Diagram used to categorize confidence levels.

**Table S1.** Description of the models used to project species distribution, growth, or biomass.

**Table S2.** Models applied to each species.

**Table S3** (provided as a separate excel file). Numerical results of the changes in abundance or growth in species or combination of species.

**Table S4.** Change in the location of centroid of the fish species.

**Table S5**. Table of the scoring of the confidence in model output for each fish species, model, and region combination presented in Fig. 1.

**References**

**Figure S1**: IPCC-based diagram between agreement and evidence used to build a qualitative scale for confidence in the models (see Methods). Results are given in Fig.1 and Table S5. Very low, and very high values were not obtained in the agreement x evidence scale of 1 to 9 (Table S5), the models confidence score ranged from 2.25 to 6.25 so only three levels are used in the results, corresponding to *(low), **(medium), and ***(high).


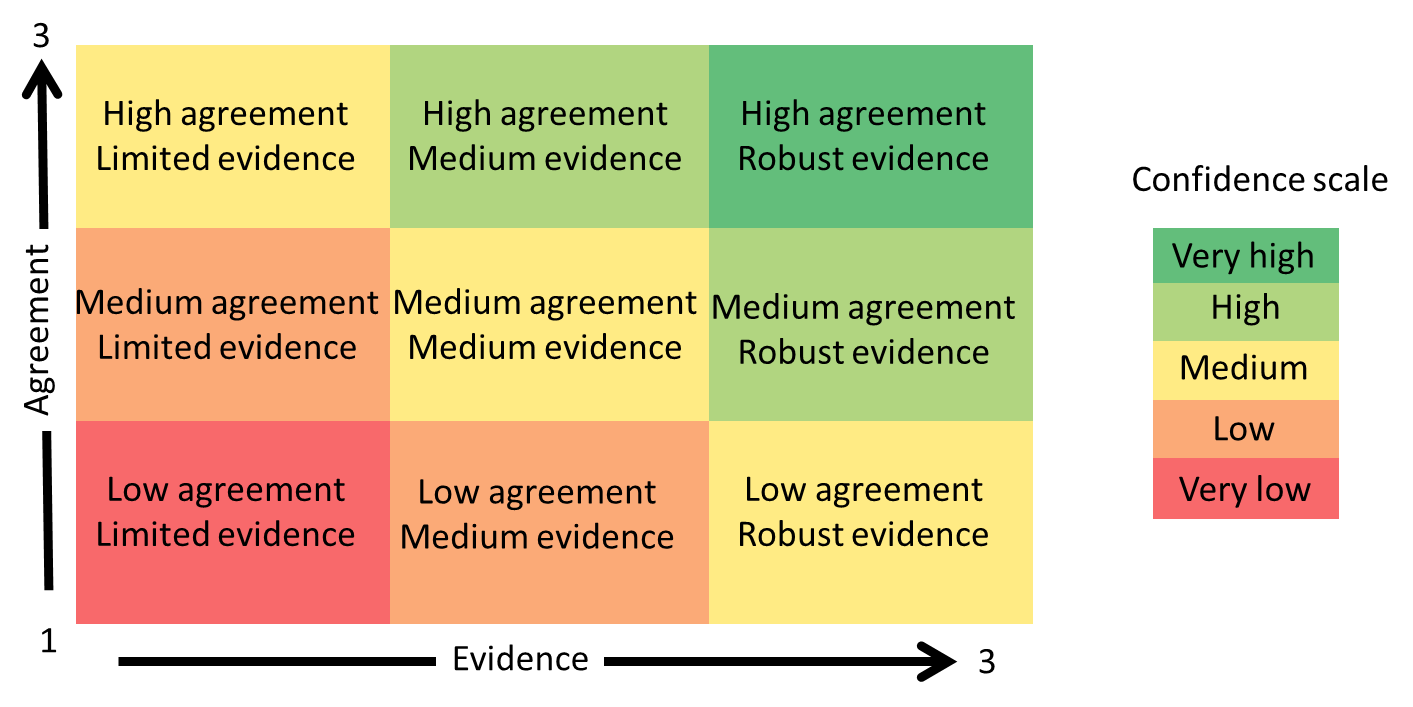


**

***

*

**Table S1**: Description of the models used to project species distribution, growth, or biomass. References of applications using these configurations are supplied. As well as where to find the model code and the outputs for this study

|  | Model name | Spatial Domain | Forcing | Type of model | Use in analysis | Reference | Code | Outputs |
| --- | --- | --- | --- | --- | --- | --- | --- | --- |
| 1 | INLA | North Sea | POLCOMS-ERSEM | Statistical Single species | Biomass | 1, 2 | DOI: [10.5281/zenodo.14998426](https://doi.org/10.5281/zenodo.14998426)  Results can be replicated with code and data in link provided | |
| 2 | Species Distribution Model | North Sea | POLCOMS-ERSEM | Statistical (habitat suitability) Single species | Distribution | 3 | For a full description of the SDM models used to describe future habitat suitability in North Sea fish species (SDM-Habitat), see Townhill et al. (2023, ref. 3). Data concerning the model results on habitat suitability for North Sea cod, saithe, herring, horse mackerel, mackerel, sardine, plaice, sole, hake, haddock, anchovy and red mullet (and 37 further North Sea species) for future decades of the 21st century, under emission scenarios RCP4.5 and RCP8.5, are available to download from the Cefas Data Hub at: <https://doi.org/10.14466/CefasDataHub.138> | |
| 3 | W Med-Small Pelagics | NW Mediterranean Sea | POLCOMS-ERSEM | Statistical (GAM) Single species | Distribution | 4 | <https://doi.org/10.5281/zenodo.14755161>  Results can be replicated with code and data in link provided | |
| 4 | W Med-Dolphinfish | NW Mediterranean Sea | POLCOMS-ERSEM | Statistical (Bayesian hierarchical model) Single species | Reproduction and growth | 5, 6 | <https://doi.org/10.6084/m9.figshare.13095638>  Results can be replicated with code and data in link provided | |
| 5 | IEO-Tuna | Western Mediterranean Sea | POLCOMS-ERSEM | Statistical Single species | Reproduction and growth | 7,8 | <https://doi.org/10.1093/icesjms/fsy135> Results can be replicated with code and data in link provided | |
| 6 | Aegean-GAM | Aegean Sea | POLCOMS-ERSEM | Statistical (GAM) Single species | Distribution | 8 | <https://cloudfs.hcmr.gr/index.php/s/AmN9FM1P1j84NS5> Results can be replicated with code and data in link provided | |
| 7 | Aegean-MaxEnt | Aegean Sea | POLCOMS-ERSEM | Statistical (MaxEnt) Single species | Distribution | 8, 9, 10 | <https://cloudfs.hcmr.gr/index.php/s/AmN9FM1P1j84NS5> Results can be replicated with code and data in link provided | |
| 8 | Baltic Atlantis | Baltic Sea | RCO-SCOBI | Mechanistic Multi-species | Biomass | 11, 12 | [https://research.csiro.au/atlantis/home/links/](https://gbr01.safelinks.protection.outlook.com/?url=https%3A%2F%2Fresearch.csiro.au%2Fatlantis%2Fhome%2Flinks%2F&data=05%7C02%7Csesa%40pml.ac.uk%7C6da9e7a7ce984c58732708dd3c506595%7C0fa5013d5a9845f0aa857dbe11f85391%7C0%7C0%7C638733038218928078%7CUnknown%7CTWFpbGZsb3d8eyJFbXB0eU1hcGkiOnRydWUsIlYiOiIwLjAuMDAwMCIsIlAiOiJXaW4zMiIsIkFOIjoiTWFpbCIsIldUIjoyfQ%3D%3D%7C0%7C%7C%7C&sdata=GnEdzSsb2%2BYDqe1nqTVKE8slQzQxICfU1GpptNCRnzs%3D&reserved=0) | [https://doi.org/10.11583/DTU.28443596](https://gbr01.safelinks.protection.outlook.com/?url=https%3A%2F%2Fdoi.org%2F10.11583%2FDTU.28443596&data=05%7C02%7Csesa%40pml.ac.uk%7C4a3245ac5c404c0f263e08dd5189891a%7C0fa5013d5a9845f0aa857dbe11f85391%7C0%7C0%7C638756373332204585%7CUnknown%7CTWFpbGZsb3d8eyJFbXB0eU1hcGkiOnRydWUsIlYiOiIwLjAuMDAwMCIsIlAiOiJXaW4zMiIsIkFOIjoiTWFpbCIsIldUIjoyfQ%3D%3D%7C0%7C%7C%7C&sdata=K%2BrFPsaXO%2FMZCOKb6vr3qws1Qogondaj2DwqGIANV%2BY%3D&reserved=0) |
| 9 | NoBa Atlantis | Nordic and Baltic Seas | ROMS | Mechanistic Multi-species | Biomass | 13–16 | [https://research.csiro.au/atlantis/home/links/](https://gbr01.safelinks.protection.outlook.com/?url=https%3A%2F%2Fresearch.csiro.au%2Fatlantis%2Fhome%2Flinks%2F&data=05%7C02%7Csesa%40pml.ac.uk%7C6da9e7a7ce984c58732708dd3c506595%7C0fa5013d5a9845f0aa857dbe11f85391%7C0%7C0%7C638733038218928078%7CUnknown%7CTWFpbGZsb3d8eyJFbXB0eU1hcGkiOnRydWUsIlYiOiIwLjAuMDAwMCIsIlAiOiJXaW4zMiIsIkFOIjoiTWFpbCIsIldUIjoyfQ%3D%3D%7C0%7C%7C%7C&sdata=GnEdzSsb2%2BYDqe1nqTVKE8slQzQxICfU1GpptNCRnzs%3D&reserved=0) | <https://doi.org/10.21335/NMDC-474993428> |
| 10 | 0D-DEB-IBM | Bay of Biscay | POLCOMS-ERSEM | Mechanistic (DEB) Single species | Biomass | 17 | [https://doi.org/10.5281/zenodo.15022036](https://gbr01.safelinks.protection.outlook.com/?url=https%3A%2F%2Fdoi.org%2F10.5281%2Fzenodo.15022036&data=05%7C02%7Csesa%40pml.ac.uk%7C6f7e5acdf9624c70c67e08dd627c88be%7C0fa5013d5a9845f0aa857dbe11f85391%7C0%7C0%7C638775009194623695%7CUnknown%7CTWFpbGZsb3d8eyJFbXB0eU1hcGkiOnRydWUsIlYiOiIwLjAuMDAwMCIsIlAiOiJXaW4zMiIsIkFOIjoiTWFpbCIsIldUIjoyfQ%3D%3D%7C0%7C%7C%7C&sdata=BInZ%2BpleoYREqidsVKMMDS8oE%2FKBq6Qeqg5ViuT5JSU%3D&reserved=0) Results can be replicated with code and data in link provided | |
| 11 | SS-DBEM | Northeast Atlantic and  Mediterranean Sea | POLCOMS-ERSEM | Mechanistic Multi-species | Biomass and distribution | 18 | <https://doi.org/10.5281/zenodo.7548113> | <https://doi.org/10.24381/cds.39c97304> |

**Table S2**: Models applied to each species (codes in Table S1), and generic group name used in Figure 2.

| Common name | Scientific name | Group | Model code |
| --- | --- | --- | --- |
| Anchovy | *Engraulis encrasicolus* | Small pelagic | 3; 10; 11 |
| Atl. Horse mackerel | *Trachurus trachurus* | Mid/large pelagic | 11 |
| Bluefin tuna | *Thunnus thynnus* | Mid/large pelagic | 5; 11 |
| Capelin | *Mallotus villosus* | Small pelagic | 9; 11 |
| Cod | *Gadus morhua* | Demersal | 2; 9; 8; 11 |
| Dolphinfish | *Coryphaena hippurus* | Mid/large pelagic | 4; 11 |
| European sprat | *Spattus sprattus* | Small pelagic | 8; 11 |
| Haddock | *Melanogrammus aeglefinus* | Demersal | 2; 11 |
| Hake | *Merluccius merluccius* | Demersal | 2; 6;7; 11 |
| Atlantic herring | *Clupea harengus* | Small pelagic | 2; 8; 9; 11 |
| Mackerel | *Scomber scombrus* | Mid/large pelagic | 2; 11 |
| Plaice | *Pleuronectes platessus* | Flatfish | 1; 2; 11 |
| Red Mullet | *Mullus barbatus* | Demersal | 6; 11 |
| Saithe | *Pollachius virens* | Demersal | 2; 11 |
| Sardine | *Sardina pilchardus* | Small pelagic | 11 |
| Sardinella | *Sardinella aurita* | Small pelagic | 3 |
| Sole | *Solea solea* | Flatfish | 2; 11 |

**Table S3:** Numerical results of the changes in abundance or growth in species or combination of species. Table provided in Excel.

**Table S4:** Change in the location of centroid of the fish species in the North Sea, NE Atlantic and Mediterranean (Med.) Sea by 2100 compared to 2000-2010 expressed in km. RCP 4.5 left, RCP 8.5 right, in a no fishing scenario. Note, only the main direction of change is shown which is North-South in the NE Atlantic and North Sea, while it is East-West in the Mediterranean Sea. Results from the SS-DBEM were used to calculate the change in population centroid.

|  | **RCP 4.5** |  | Species |  | **RCP 8.5** |  |
| --- | --- | --- | --- | --- | --- | --- |
| Med. Sea  (heading, km) | NE Atlantic (heading, km) | North Sea (heading, km) |  | North Sea (heading, km) | NE Atlantic (heading, km) | Med. Sea (heading, km) |
| - | - | S, 13 | **Cod** | S, 33 | - | - |
| - | - | S, 12 | **Saithe** | S, 32 | - | - |
| - | N, 233 | N, 22 | **Herring** | N, 20 | N, 437 | - |
| W, 53 | N, 64 | N, 20 | **Atl. Horse Mackerel** | N, 32 | N, 169 | W, 79 |
| W, 90 | N, 138 | N, 32 | **Mackerel** | N, 50 | N, 309 | W, 554 |
| W, 645 | N, 106 | N, 37 | **Sardine** | N, 60 | N, 249 | E, 723 |
| - | S, 58 | S, 4 | **Plaice** | S, 44 | S, 133 | - |
| E, 11 | N, 29 | S, 1 | **Sole** | S, 3 | N, 22 | W, 44 |
| E, 447 | N, 114 | S, 3 | **Hake** | S, 17 | N, 116 | E, 416 |
| - | N, 3 | S, 9 | **Haddock** | S, 27 | N, 25 | - |
| W, 124 | N, 52 | N, 64 | **Anchovy** | N, 92 | N, 116 | W, 579 |
| W, 786 | - | - | **Dolphinfish** | - | - | W, 819 |
| W, 2 | N, 7 | - | **Tuna** | - | N, 44 | W, 41 |
| W, 6 | N, 72 | S, 3 | **Red Mullet** | S, 7 | N, 49 | W, 21 |

**Table S5:** Scoring of the confidence in each model outputs depending on the fish species. Agreement was evaluated for projections from two or more models for the same species in the same region, looking at agreement for both the direction and strength of the trend. When only one model was applied, it was scored based on whether multiple scenarios were explored and the extent to which the model was state-of-the-art or newly implemented. Evidence was based on the extent of agreement between the extant validation data (historical and present-day field data) as well as past model validation exercises where available. Both evidence and agreement were scored on a scale from 1 (poor) to 3 (very good), if in doubt, a lower score was chosen. The scores were multiplied, giving a final score on a scale of 1 to 9, with 5 categories of very low (1+), low (2+), medium (3+), high(6+) and very high confidence (9). The final score of the model had them range from low (*) to high confidence (***) levels.

|  | Confidence scoring | | | | |
| --- | --- | --- | --- | --- | --- |
| Fish species | Agreement | Evidence | Final | Scale | |
| Capelin | 1.5 | 2 | 3 | Medium | ** |
| Norwegian herring | 1.5 | 2 | 3 | Medium | ** |
| Sprat | 1.5 | 2 | 3 | Medium | ** |
| Cod (Norwegian, Barents, and Baltic Seas) | 1.5 | 2 | 3 | Medium | ** |
| Cod (North Sea) | 3 | 2 | 6 | High | *** |
| Saithe | 2.5 | 1.5 | 3.75 | Medium | ** |
| Herring | 2.5 | 1.5 | 3.75 | Medium | ** |
| Atl. Horse Mackerel | 1.5 | 2.5 | 3.75 | Medium | ** |
| Mackerel | 1.5 | 1.5 | 2.25 | Low | * |
| Sardine | 1.5 | 1.5 | 2.25 | Low | * |
| Plaice | 3 | 2 | 6 | High | *** |
| Sole | 2 | 2 | 4 | Medium | ** |
| Hake | 2.5 | 1.5 | 3.75 | Medium | ** |
| Hake (Aegean Sea) | 3 | 2 | 6 | High | *** |
| Haddock | 3 | 1.5 | 4.5 | Medium | ** |
| Anchovy (SS-DBEM) | 2 | 2 | 4 | Medium | ** |
| Anchovy (DEB-IDM) | 1.5 | 2 | 3 | Medium | ** |
| Sardinella | 1.5 | 2 | 3 | Medium | ** |
| Dolphinfish | 2.75 | 3 | 8.25 | High | *** |
| Bluefin Tuna | 2 | 2.5 | 5 | Medium | ** |
| Red mullet | 2.5 | 2.5 | 6.25 | High | *** |

**References**

1. Rue, H., Martino, S. & Chopin, N. *Approximate Bayesian inference for latent Gaussian models by using integrated nested Laplace approximations*. *J. R. Statist. Soc. B* vol. 71 https://academic.oup.com/jrsssb/article/71/2/319/7092907 (2009).

2. Zuur, A., Ieno, E. N. & Saveliev, A. A. *Spatial, temporal and spatial-temporal ecological data analysis with r- inla*. vol. 1 (Highland Statistics Ltd, 2017).

3. Townhill, B. L., Couce, E., Tinker, J., Kay, S. & Pinnegar, J. K. Climate change projections of commercial fish distribution and suitable habitat around north western Europe. *Fish and Fisheries* **24**, 848–862 (2023).

4. Maynou, F., Sabatés, A., Ramirez-Romero, E., Catalán, I. A. & Raya, V. Future distribution of early life stages of small pelagic fishes in the northwestern Mediterranean. *Clim Change* **161**, 567–589 (2020).

5. Moltó, V. *et al.* Projected effects of ocean warming on an iconic pelagic fish and its fishery. *Sci Rep* **11**, (2021).

6. Rambo, H., Ospina-Álvarez, A., Catalán, I. A., Maynou, F. & Stelzermüller, V. Unravelling the combined effects of socio-political and climate change scenarios for an artisanal small-scale fishery in the Western Mediterranean. *Ecology and Society* **27**, (2021).

7. Reglero, P. *et al.* Pelagic habitat and offspring survival in the eastern stock of Atlantic bluefin tuna. *ICES Journal of Marine Science* **76**, (2019).

8. Peck, M. A. *et al.* *Climate Change and European Fisheries and Aquaculture: ‘CERES’’ Project Synthesis Report’*. (2020).

9. Damalas, D., Sgardeli, V., Vasilakopoulos, P., Georgios Tserpes, | & Maravelias, C. Evidence of climate-driven regime shifts in the Aegean Sea’s demersal resources: A study spanning six decades. *Ecol Evol* **11**, 16951 (2021).

10. Phillips, S. B., Aneja, V. P., Kang, D. & Arya, S. P. Modelling and analysis of the atmospheric nitrogen deposition in North Carolina. in *International Journal of Global Environmental Issues* vol. 6 231–252 (Inderscience Publishers, 2006).

11. Bossier, S. *et al.* Integrated ecosystem impacts of climate change and eutrophication on main Baltic fishery resources. *Ecol Modell* **453**, 109609 (2021).

12. Bossier, S. *et al.* The Baltic Sea Atlantis: An integrated end-to-end modelling framework evaluating ecosystem-wide effects of human-induced pressures. (2018) doi:10.1371/journal.pone.0199168.

13. Hansen, C., Nash, R. D. M., Drinkwater, K. F. & Hjøllo, S. S. Management Scenarios Under Climate Change – A Study of the Nordic and Barents Seas. *Front Mar Sci* **6**, (2019).

14. Hansen, C., Skern-Mauritzen, M., van der Meeren, G., Jähkel, A. & Drinkwater, K. *Set-up of the Nordic and Barents Seas (NoBa) Atlantis model*. (2016).

15. Skogen, M. D., Paul Budgell, W. & Rey Skogen, F. Interannual variability in Nordic seas primary production. *ICES Journal of Marine Science* **64**, 889–898 (2007).

16. Sandø, A. B. *et al.* Downscaling IPCC control run and future scenario with focus on the Barents Sea. *Ocean Dyn* **64**, 927–949 (2014).

17. Bueno-Pardo, J., Petitgas, P., Kay, S. & Huret, M. Integration of bioenergetics in an individual-based model to hindcast anchovy dynamics in the Bay of Biscay. *ICES Journal of Marine Science* **77**, (2020).

18. Fernandes, J. A. *et al.* Can we project changes in fish abundance and distribution in response to climate? *Glob Chang Biol* **26**, 3891–3905 (2020).
